# Virologic failure in HIV-positive adolescents with perfect adherence in Uganda: a cross-sectional study

**DOI:** 10.1186/s41182-019-0135-z

**Published:** 2019-01-17

**Authors:** Julian Natukunda, Peter Kirabira, Ken Ing Cherng Ong, Akira Shibanuma, Masamine Jimba

**Affiliations:** 1 Public Health and Management, Institute of Health, International Health Sciences University, Kampala, Uganda; 2 Department of Community and Global Health, Graduate School of Medicine, The University of Tokyo, Tokyo, Japan

**Keywords:** Human immunodeficiency virus (HIV), Adherence, Adolescents, Viral suppression, Virologic failure, Antiretroviral therapy

## Abstract

**Background:**

Adolescents living with human immunodeficiency virus (HIV) die owing to acquired immune deficiency syndrome (AIDS)-related causes more than adults. Although viral suppression protects people living with HIV from AIDS-related illnesses, little is known about viral outcomes of adolescents in sub-Saharan Africa where the biggest burden of deaths is experienced. This study aimed to identify the factors associated with viral load suppression among HIV-positive adolescents (10–19 years) receiving antiretroviral therapy (ART) in Uganda.

**Methods:**

We conducted a cross-sectional study among school-going, HIV-positive adolescents on ART from August to September 2016. We recruited 238 adolescents who underwent ART at a public health facility and had at least one viral load result recorded in their medical records since 2015. We collected the data of patients’ demographics and treatment- and clinic-related factors using existing medical records and questionnaire-guided face-to-face interviews. For outcome variables, we defined viral suppression as < 1000 copies/mL. We used multivariate logistic regression to determine factors associated with viral suppression.

**Results:**

We analyzed the data of 200 adolescents meeting the inclusion criteria. Viral suppression was high among adolescents with good adherence > 95% (adjusted odds ratio [AOR] 2.73, 95% confidence interval [95% CI, 1.09 to 6.82). However, 71% of all adolescents who did not achieve viral suppression were also sufficiently adherent (adherence > 95%). Regardless of adherence status, other risk factors for viral suppression at the multivariate level included having a history of treatment failure (AOR 0.26, 95% CI, 0.09 to 0.77), religion (being Anglican [AOR 0.19, 95% CI, 0.06 to 0.62] or Muslim [AOR 0.17, 95% CI, 0.05 to 0.55]), and having been prayed for (AOR 0.38, 95% CI, 0.15 to 0.96).

**Conclusion:**

More than 70% of adolescents who experienced virologic failure were sufficiently adherent (adherence > 95). Adolescents who had unsuppressed viral loads in their initial viral load were more likely to experience virologic failure upon a repeat viral load regardless of their adherence level or change of regimen. The study also shows that strong religious beliefs exist among adolescents. Healthcare provider training in psychological counseling, regular and strict monitoring of adolescent outcomes should be prioritized to facilitate early identification and management of drug resistance through timely switching of treatment regimens to more robust combinations.

**Electronic supplementary material:**

The online version of this article (10.1186/s41182-019-0135-z) contains supplementary material, which is available to authorized users.

## Introduction

In many countries, the “treat all” approach is implemented to identify HIV-positive people earlier and get them on treatment. Its goal is to suppress viral replication by maintaining high levels of adherence to antiretroviral therapy (ART). These efforts are part of the global response toward achieving “the third 90” in the 90-90-90 targets, an initiative to end the AIDS epidemic as a public health threat by 2030.

Briefly, the Joint United Nations Program on HIV and AIDS’ 90-90-90 campaign aims to have 90% of people living with HIV to know their status, have 90% of people living with HIV who know their status start or maintain their treatment, and have 90% of people on treatment to be virally suppressed by 2020. Achieving viral suppression protects people living with HIV from acquired immune deficiency syndrome (AIDS)-related illnesses and lowers the risk of transmission to others. In 2016, however, only 44% of the people living with HIV who were on treatment had viral suppression globally [1].

To improve viral suppression, adolescents should be more cared for. This is because, globally, up to 150 adolescents die every day due to AIDS-related illnesses. In 2016, 91% of adolescent deaths worldwide were reported in sub-Saharan Africa, and the rate of AIDS-related deaths among this age group have not reduced [2, 3]. In addition, children and adolescents from different parts of the world had worse viral suppression outcomes than adults [4–6].

So far, barriers to and factors that promote viral suppression have been identified. Commonly cited barriers among adolescents include pill burden, medication taste, secrecy/stigma, drug toxicity and resistance, clinic-related factors, being sick, missed appointments, loss of a mother, strong religious beliefs, and poor knowledge about HIV, among others [7–11]. On the contrary, promoting factors have also been identified: use of community interventions [12], setting up adolescent-specific care spaces [13], and maintaining adherence > 95% [4]. However, most of the recommendations from these studies require financial support, which is scarcely available in low-income countries, and interventions may collapse once support is withdrawn. Therefore, more cost-effective interventions are needed.

Uganda ranked among the top 20 high burden countries contributing 5% of AIDS-related deaths among adolescents in 2014 [14]. The deaths are attributed to the late diagnosis and poor access to treatment with most perinatally infected children starting treatment later in life. Like other countries, Uganda committed to achieve the 90-90-90 targets by 2020; however, the country is still short on achieving the ambitious target with the third 90 scoring lowest along the cascade. In 2017, about 73% of the people living with HIV knew their HIV status; 67% were enrolled on ART; while almost 60% had achieved viral suppression [15]. To identify people living with HIV who are likely to fail on treatment (including adolescents) and monitor their quality of life, the Uganda Ministry of Health recommended viral load testing for all people who are receiving ART for at least 6 months or more. Children under 15 years continued to have much lower rates of viral suppression compared to adults [4, 15, 16].

Although adherence to ART is important for viral suppression, some children and adolescents are sufficiently adherent but their viral loads remain high, while others have suppressed viral loads despite poor adherence [17–19]. In Uganda, information on viral suppression among adolescents aged 10–19 years is limited. This is because the age disaggregation groups adolescents 10–15 years with children aged 0–14 years while those 16–19 years are considered among adults. It is therefore difficult to ascertain outcomes of adolescents on ART with the current data [15]. This study aimed to identify the factors associated with viral suppression among adolescents aged 10–19 years and characteristics of sufficiently adherent adolescents who fail to achieve viral suppression.

## Materials and methods

### Study design

We conducted a cross-sectional study of HIV-positive adolescents receiving ART at Jinja Regional Referral Hospital (RRH), Uganda, from August to September 2016. To assess the individual and socio-demographic factors associated with viral suppression, we conducted face-to-face interviews using semi-structured questionnaires. Additionally, using a data extraction tool developed in Excel, we collected secondary (retrospective) data on ART-related factors such as duration of ART, CD4 count at initiation, adherence status (past 12 months), treatment failure, World Health Organization clinical staging, and nutritional status. We developed the data extraction tool based on screening key indicators monitored nationally using the individual medical card, viral load register, and electronic database.

### Study area

We conducted this study at Jinja Regional Referral Hospital—a public referral center for complicated cases and specialized laboratory tests in the East Central region, Uganda. The hospital runs a separate wing for children, pediatrics, and adolescents. Adolescents living with HIV aged 18 years are transferred to the adult HIV clinic located 3 m away from the main hospital. Health workers regularly offer pre-regimen and adherence counseling, discuss appointment schedules, probe for other illnesses, and explain viral load outcomes to adolescents and caretakers at every clinical review appointment.

In June 2016, over 600 adolescents at Jinja RRH were given ART. Of them, 397 adolescents were actively attending their scheduled clinical review appointments; only 238 adolescents had access to a viral load test. Mass viral load monitoring using viral load testing begun in 2015 at this hospital. Using the viral load national testing algorithm, children and adolescents below 20 years are required to have a viral load done every 6 months [20]. The study period coincided with school holidays where adolescents could be easily seen, and appointments had been scheduled to coincide with the adolescent clinic day.

### Study participants

HIV-positive adolescents (10–19 years) who received ART for at least 6 or more months at Jinja RRH and had viral load results in their medical records were included in this study. The viral load results considered in this study were those documented between 1 January 2015 to 31 July 2016. We obtained assent from the caretakers of adolescents aged 10–14 years and consent from adolescents aged 15–19 years. We selected 238 adolescents to participate in the study from the total eligible adolescents enrolled on ART using Open Medical Records System (OpenMRS), an electronic database.

### Sample size calculation

Given the total population of 600 HIV-positive adolescents, 397 adolescents (10–19 years) were receiving ART at Jinja RRH’s Nalufenya children’s and adult HIV clinic. Of the 397 adolescents, 238 had accessed a viral load test in the past 2 years.

Using Sloven’s formula

$$ n=\frac{N}{\left(1+{\mathrm{Ne}}^2\right)} $$

The total number of adolescents required for our study was approximately 209. We therefore decided to study all the adolescents, whose files contained viral load results, representing 60% of the study target population.

### Data collection

The primary outcome was viral suppression defined as viral load < 1000 copies/mL [21]. We collected individual viral load results and suppression status from the viral load result forms kept in the medical files. We used the most recent viral load results for adolescents (results documented between January 2015 and July 2016). For other treatment-related factors, we used data from individual ART cards and electronic medical records (OpenMRS) software. For example, we extracted data on adherence status from the ART cards, which tracked the adherence of each adolescent for the past 12 months before their latest viral load test. For adolescents who were on ART for less than 12 months, we calculated the adherence rate based on the total number of months spent on treatment (at least 6 months). Medication adherence in this study was referred to as the degree to which adolescents took their prescribed medicines (includes dosage, time of the day) using pill counts as a primary measure. Adolescents’ adherence was described as good, fair, or poor based on the following; G (good) > 95%, F (fair) if between 85% and 94%, and P (poor) < 85% [20]. According to the ART card summary guide (at the bottom of the generic ART card), the level of adherence was estimated using the number of missed doses per month based on a twice daily regimen. For example, if an adolescent missed 3 or less doses in a month, adherence was documented as good; if 4–8 doses were missed, it would be recorded as fair adherence, and 9 or more missed doses implied poor adherence. Other variables from ART cards included age, sex, regimen type [20], CD4 count, appointment keeping, history of treatment failure, nutritional status, clinical stage, opportunistic infection, treatment start date, and counseling after treatment [4, 20].

#### Sociodemographic variables

We collected information about age and sex using face-to-face questionnaires to cross examine information collected from the medical cards. In addition, we asked questions about level of education, religion, being prayed for, believing in God or Allah healing their illness, having both parents alive, perceived family support [9, 22], level of satisfaction of health services offered, convenience of scheduled appointments, and staying with a family member who is HIV positive [23, 24].

We recruited eight field assistants (particularly those working within the HIV clinic) to collect data and trained them on how to fill the data tools, resolve issues related to obtaining consent, and perform procedures according to ethical standards. The data assistants collected information from the patient cards into a data extraction tool. They then conducted face-to-face interviews and filled out questionnaires as they interacted with adolescents and their care givers during scheduled clinical review appointments. Whereas the field assistants were conversant with the local dialect, they had no formal connections with the respective adolescents, which minimized any bias in responses to the subjects. In addition, while extensive training was offered to the field assistants to fully acquaint them with the data collection procedures, they were ignorant about the study outcome. The counselors further contacted caregivers of adolescents whose appointments had passed or were scheduled on a future date to come with their children to the clinic using phone calls. We validated the data abstraction tool and pretested semi-structured questionnaires before the study.

### Data analysis

We coded and entered quantitative data from study tools into a predesigned Excel spreadsheet and performed statistical analyses using STATA version 13.1 (College Station, TX, USA). We also performed descriptive analyses and summarized data on continuous variables such as age, viral load, and mean and their corresponding standard deviations. Then, we summarized the categorical variables including sex, viral suppression, and religion, among others, as proportions. For binary outcomes, we obtained odds ratio for each independent variable, adjusting for suppression. We performed multiple logistic regression analyses and chi-square tests to determine any possible association at 95% confidence level. Since CD4 count at initiation had missing baseline values, we performed multiple imputations (10 imputations) using variables associated with the outcome variable. While we did not have a command for variance inflation factor in stata in the case of multiple imputation, we checked variance inflation factor without multiple imputation and did not find multicollinearity. We included significant (*P* ≤ 0.05) variables and other variables that were not considered significant but were reported to influence viral outcomes in the final model [4, 9, 13]. Finally, we included all variables with *P* value < 0.05 from our multivariable analysis as independent predictors of viral suppression in this population.

### Ethical considerations

We obtained a waiver from the International Health Sciences University’s Degree Research and Ethics Committee. The reason for the waiver was that the study had no potential to cause harm whether physical or psychological to the participants. The decision was based on the National Guidelines for Research involving humans as research participants developed by the Uganda National Council for Science and Technology. We also obtained permission from the hospital administration to conduct the study. We sought an informed consent for adolescents aged 15 years and above, or informed assent from parents/caretakers of adolescents aged below 15 years. To ensure confidentiality, we assigned study participants with unique identifiers.

## Results

### General characteristics

Table 1 shows the basic characteristics of adolescents. Overall, 200 adolescents participated in the study. The mean age was 13.2 (standard deviation: 2.4) years, and 52% of them were male adolescents. Of the 200 adolescents, 131 (65.5%) achieved viral suppression. Over 75% of adolescents attended primary level education and received support from their family members such as accommodation, feeding, and education. The HIV status of nearly 80% of the adolescents was known among family members. About 65% of adolescents lived with at least one family member of the same HIV status in one household. Adolescents belonged to either one of the following religious denominations: Catholic, Anglican, Muslim, and others. In addition, 75% of the adolescents attended clinic services in the same space as adults and believed in God healing their illness.

**Table 1:** Socio-demographic and client-related characteristics of adolescents (*n* = 200)

Characteristics		*n*	%
Age mean (standard deviation, SD)		13.2 (2.4)	
Viral suppression	Yes	131	65.5
	No	69	34.5
Sex	Male	104	52.0
	Female	96	48.0
Education	Primary	151	75.5
	Ordinary	39	19.5
	Advanced	4	2.0
	Tertiary	3	1.5
	No education	3	1.5
Perceived family support	Yes	162	81.0
	No	38	19.0
Other family member with HIV	Yes	133	66.5
	No	67	33.5
Adolescent attending clinic in the same space as adults	Yes	148	74.0
	No	52	26.0
Religion	Catholic	47	23.5
	Anglican	64	32.0
	Muslim	59	29.5
	Other	30	15.0
Ever been prayed for	Yes	95	47.5
	No	105	52.5
Believe in God healing their illness	Yes	149	74.5
	No	51	25.5
Both parents alive	Yes	83	41.0
Disclosure among family members	No	117	59.0
	Yes	158	79.0
	No	42	21.0

Table 2 presents the univariate (regimen-and treatment-related) characteristics of adolescents. Over 50% had been on treatment for 5 or more years while over 80% had good adherence above 95%, had never experienced treatment failure and belonged to clinical stage 1. Overall, about 90% adolescents had a normal nutritional status.

**Table 2:** Univariate treatment characteristics of adolescents

Characteristics		*n*	%
Years on ART (years)	0–2	35	17.5
	3–5	59	29.5
	More than 5	106	53.0
CD4 count at initiation (cells/mL)	More than 500	54	27.0
	200–499	45	22.5
	Less than 200	43	21.5
	Missing	58	29.0
Adherence status	Good > 95%	160	80.0
	Fair/poor < 95%	40	20.0
History of treatment failure	Yes	29	14.5
	No	171	85.5
WHO clinical stage	I	143	86.1
	II	6	3.6
	III	15	9.1
	IV	2	1.2
Nutritional status	Normal	150	89.8
	MAM	13	7.8
	SAM	4	2.4
Regimen	AZT + 3TC + EFV	76	38.0
	AZT + 3TC + NVP	116	58.0
	ABC + 3TC + EFV	8	4.0

Table 3 shows the regimen and health-related characteristics of 200 adolescents with or without viral suppression. Both in the suppression and non-suppression groups, more than 50% of adolescents spent at least 5 or more years on ART and were started on the same regimen (AZT + 3TC + NVP). Over 70% of adolescents had good adherence (> 95%) and no history of treatment failure. About 85% of adolescents had WHO clinical stage I, while more than 90% had normal nutritional status. However, CD4 counts of the adolescents at the start of ART varied largely between the two groups. For the suppression group, the proportion of adolescents who achieved viral suppression increased with increasing CD4 counts at ART initiation [16% (CD4 < 200 cells), 23% (CD4 200–499 cells), and 34% (CD4 > 500 cells], while more adolescents in the non-suppression group tended to have lower CD4 counts at ART initiation [30% (CD4 < 200 cells), 22 (CD4; 200–499 cells), and 15% (CD4 > 500 cells)].

**Table 3:** Treatment- and health-related characteristics of adolescents and viral suppression

Characteristics		Achieved viral suppression (*n* = 131)		No viral suppression (*n* = 69)	
		*n*	%	*n*	%
Viral suppression	Men	63	48.0	41	59.0
	Women	68	52.0	28	41.0
Years on ART (years)	0–2	28	21.4	7	10.1
	3–5	36	27.5	23	33.3
	More than 5	67	51.1	39	56.5
CD4 count at initiation (cells/mL)	More than 500	44	33.6	10	14.5
	200–499	30	22.9	15	21.7
	Less than 200	22	16.8	21	30.4
	Missing	35	26.7	23	33.3
Adherence status	Good > 95%	111	84.7	49	71.0
	Fair/poor < 95%	20	15.3	20	29.0
History of treatment failure	Yes	10	7.6	19	27.5
	No	121	92.4	50	72.5
WHO clinical stage	I	116	88.5	58	84.1
	II	3	2.3	3	4.3
	III	8	6.1	8	11.6
	IV	4	3.1		
Nutritional status	Normal	118	90.0	65	94.2
	MAM	9	6.9	4	5.8
	SAM	4	3.1		
Regimen	AZT + 3TC + EFV	7	35.9	29	42.0
	AZT + 3TC + NVP	77	58.8	39	56.5
	ABC + 3TC + EFV	7	5.3	1	1.5

Notes: *SAM* severely acutely malnourished, *MAM* moderately acutely malnourished

Table 4 shows the factors associated with viral suppression among adolescents when all variables were regressed. At multivariate level, an adherence rate of > 95% (AOR 2.73, 95% CI 1.09 to 6.82) was associated with viral suppression. Adolescents were less likely to achieve viral suppression if they belonged to the following categories: those who had a history of treatment failure (AOR 0.26, 95% CI 0.09 to 0.77) and had normal nutritional status (AOR 0.11, 95% CI 0.02 to 0.67). In addition, being Anglican (AOR 0.19, 95% CI 0.06 to 0.62) or Muslim (AOR 0.17, 95% CI 0.05 to 0.55) and having ever been prayed for (AOR = 0.38, *P* < 0.05) were risk factors of viral suppression.

**Table 4:** Factors associated with viral suppression among adolescents on ART

Variables		Adjusted model		
		AOR	CI	*P* value
Age		1.01	0.82–1.23	0.95
Sex	Male	1.00		
	Female	1.49	0.72–3.08	0.28
Education level	Primary	1.00		
	Ordinary, advanced, and tertiary	0.65	0.24–1.81	0.42
Perceived family support	No	1.00		
	Yes	1.72	0.36–8.21	0.49
Disclosure among family members	No	1.00		
	Yes	1.53	0.38–6.12	0.55
Religion	Catholic	1.00		
	Anglican	0.19	0.06–0.62	0.01
	Muslim	0.17	0.05–0.55	0.003
	Other	0.42	0.12–1.49	0.18
Years on ART	0–2 years	1.00		
	3–5 years	0.61	0.16–2.42	0.49
	More than 5 years	0.74	0.19–2.81	0.66
CD4 count at initiation	> 500 cells/mL	1.00		
	200–499 cells/mL	0.44	0.15–1.35	0.15
	< 200 cells/mL	0.27	0.07–1.02	0.05
Adherence status	Poor/Fair	1.00		
	Good	2.73	1.09–6.82	0.03
Treatment failure	No	1.00		
	Yes	0.26	0.09–0.77	0.01
WHO clinical stage	Stages I and II	1.00		
	Stages III and IV	0.65	0.17–2.45	0.52
Nutritional status	Moderate/severely malnourished	1.00		
	Normal	0.11	0.02–0.67	0.02
Regimen	AZT + 3TC + EFV	1.00		
	AZT + 3TC + NVP	1.36	0.56–3.27	0.49
	ABC + 3TC + EFV	3.22	0.28–44.06	0.33
Attending clinic in the same space with adults	No	1.00		
	Yes	0.54	0.22–1.35	0.19
Ever been prayed for	No	1.00		
	Yes	0.38	0.15–0.96	0.04
Believe in God healing their illness	No	1.00		
	Yes	2.61	0.87–7.81	0.09
Another member living with HIV in the same household	No	1.00		
	Yes	1.35	0.61–2.99	0.46

Notes: *n* = 200

Additional file 1: Table S1 shows detailed individual and treatment characteristics of 49 adolescents who were adherent (> 95%) but failed to achieve viral suppression. Of the 49 adolescents, 7 (14.3%) had experienced treatment interruptions (missed medication) at least once in 12 months. The remaining 42 adolescents had medication adherence of 100% across all the 12 months. In this group, male adolescents (63%) constituted the biggest percentage. Likewise, all the 4 (8%) adolescents who were malnourished were men and belonged to clinical stage III. A total of 34 (69%) adolescents had their CD4 counts at ART initiation documented; about 18 adolescents had CD4 < 200 cells/mL, 10 had 200–499 cells/mL, and only 6 had > 500 cells/mL. About 75% of 49 adolescents had been on treatment for 5 or more years.

## Discussion

Out of 200 adolescents, 69 (34.5%) did not achieve viral suppression. Of 69, 71% had a good adherence (above 95%). Altogether, adolescents in the study had a low viral suppression rate than recommended in the 90-90-90 targets despite having good adherence. It is possible that the number of HIV-positive people with good adherence who have poor viral outcomes is increasing, especially in developing countries where treatment options are not regularly updated. This is one of the few studies which reports sufficiently adherent adolescents with no history of treatment interruptions failing to achieve viral suppression. Some of the characteristics of these adolescents include being on a regimen containing nevirapine at initiation and having a history of treatment failure.

In this study, the viral failure rate among adolescents on ART was 34.5%. This rate is higher than that reported for low- and middle-income countries (29%), and in South Africa (19%) [5, 10]. However, a study conducted in Zimbabwe and another recently concluded study in Uganda reported slightly higher rate of viral failure (43%) among children and adolescents on ART [25, 26]. The study results which show increasing rates of HIV drug resistance (HIVDR) are in line with recent findings by WHO showing an increase in levels of HIVDR even in the context of well-managed HIV treatment programs [27].

This study found that an adherence rate of > 95% was positively associated with viral suppression, and 65.5% of adolescents achieved viral suppression. However, even when adherence was more than 95%, 71% of 69 adolescents failed to achieve viral suppression. This failure might be explained as follows: first, from the Additional file 1: Table S1, regimen-specific differences were more pronounced in adolescent males, especially those enrolled on a treatment combination consisting of nevirapine (AZT + 3TC + NVP). From the Additional file 1: Table S1, 57% of the adolescents who experienced virologic failure were on a nevirapine combination. However, apart from the skin rash, no specific adverse events were reported among adolescents on this regimen. Nevirapine use, in the past, was known to be associated with increased risk of ART failure [28, 29]. In sub-Saharan Africa, however, treatment combination options remain largely unchanged for HIV-positive people, and transition to more robust regimens is costly.

Second, the time spent on ART should be considered. Longer exposure to ART (5 years and above) was a common characteristic among adolescents who failed to achieve viral suppression. This finding is consistent with that reported in studies conducted in Northwest Ethiopia [30] and Swaziland [31]. Because long-term ART is common in perinatally infected children transitioning into young adults, viral failure may be explained by the possible accumulation of drug resistance mutations over time (adherence to a failing regimen).

Third, only 14.3% of the 49 adolescents with good adherence who failed to achieve viral suppression had experienced treatment interruptions at least once in 12 months. Missed antiretroviral doses or interruptions in therapy may result in HIV drug resistance hence virologic failure [32]. Some studies have reported low adherence for people with strong spiritual beliefs who tend to delay or miss medication during fasting, after being baptized with holy water [10], or after receiving healing prayers [11]. Although treatment interruptions were indicated by a “fair” or “poor” adherence status in the medical files, there was no specific explanation about the nature of the interruptions and how long they lasted within the month. More so, information about interruptions was obtained via individual self-reports which are subject to bias. Future research should prioritize inventing a daily measure or tool for capturing treatment interruptions.

In this study, 15% of 131 HIV-positive adolescents with poor adherence (< 95%) achieved viral suppression. This finding is consistent with those reported in other studies [17–19]. The Uganda Ministry of Health Guidelines recommend intensive adherence counseling for 6 weeks for anyone with high viral loads (> 1000 copies) and a repeat viral load test conducted after the third intensive adherence counseling session. In our study, 168 (84%) adolescents had a repeat viral load result in their files overall. Of these, 61 (78%) of the adolescents had unsuppressed viral load after a repeat viral load. In this case, intensive adherence counseling would not be a priority for such adolescents; instead, efforts should be focused on understanding facilitators of viral suppression among this group. Furthermore, many countries have a limited number of health workers trained in adolescent health services [33]. Training of all health workers in adolescent health service delivery may address existing challenges and provide better understanding of the promoting factors and barriers to suppression among this age group.

Adolescents were less likely to achieve viral suppression if they had previously failed on treatment [4], were Muslim or Anglican, and had ever been prayed for. Contrary to our expectations, WHO clinical stage, duration of treatment, CD4 count [31], family support, and sharing clinic space were not associated with viral suppression. These findings were also contrary to those of other studies conducted in Zimbabwe [13], Tanzania [22], and Ethiopia [28], which indicated that family support and establishment of adolescent-specific clinic spaces [13] improved viral outcomes.

Adolescents have been reported to have better viral outcomes when they had a person living with HIV in the same household or had been switched to a second line treatment [25, 34]. In this study, although the majority (66.5%) had members in their family living with a positive HIV status, there was no significant relationship with viral suppression. In addition, few adolescents (14%) were switched to the second line treatment despite their viral outcome status. Majority (61%) who had not achieved suppression had not been switched, and no markers were in place to prioritize for a change of regimen.

In this study, age and education (school attendance) were not significantly associated with viral load suppression. However, adolescents aged 12 and 16 years were more likely to have an unsuppressed viral load in both the initial and subsequent viral load despite consistently high levels of adherence and family support. This is contrary to the findings from other studies conducted in Uganda where adolescents who attended school were found with lower levels of adherence to treatment and older adolescents had high tendencies of missed scheduled appointments [16, 35].

This study had several limitations. Although our study was cross sectional in nature, we collected viral load results from secondary data. It is possible that the viral suppression status of some adolescents may have changed at the time of the study. We also sampled only those adolescents who had a viral load test result documented in their medical records between January 2015 and July 2016. Other limitations include limited knowledge and data about drug-resistance testing, missing data, the use of sub-optimal treatment regimens, limited sample size, and non-ascertainment of prior treatment interruptions (such as history of adherence since start of ART). For nutrition, we used the most current nutritional status of adolescents documented in the individual ART cards and could not rule out that nutritional status might have been different at time when the viral load test was taken. We restricted the sample to adolescents from the East Central region of Uganda; hence, our results could be different from those conducted in other settings. The need for caretaker’s consent was a major obstacle especially when young adolescents reported to the clinic alone.

## Conclusion

More than 70% of all adolescents who did not achieve viral suppression were sufficiently adherent (with adherence > 95%). Adolescents who had unsuppressed viral loads in their initial viral load were more likely to experience virologic failure upon a repeat viral load regardless of their adherence level or change of regimen. The study also shows that strong religious beliefs exist among adolescents. Healthcare provider training in psychological counseling, regular and strict monitoring of adolescent outcomes should be prioritized to facilitate early identification and management of drug resistance through timely switching of treatment regimens to more robust combinations.

## Additional file

Additional file 1: **Table S1.** Key characteristics of adolescents whose adherence level was good (> 95%) but failed to achieve viral suppression (*n* = 49). (DOCX 13 kb)

## Abbreviations

3TC: Lamivudine
ABC: Abacavir
AIDS: Acquired immune deficiency syndrome
AOR: Adjusted odds ratio
ART: Antiretroviral therapy
CI: Confidence interval
EFV: Efavirenz
HIV: Human immunodeficiency virus
HIVDR: HIV drug resistance
NVP: Nevirapine
OpenMRS: Open Medical Records System
RRH: Regional Referral Hospital
