# Supplementary material for: Virologic failure in HIV-positive adolescents with perfect adherence in Uganda: a cross-sectional study
Source: Trop Med Health. 2019 Jan 17;47:8. doi: 10.1186/s41182-019-0135-z (PMC6337787; doi:10.1186/s41182-019-0135-z)
Supplement: Supplementary file 1 — Table S1. Key characteristics of adolescents whose adherence level was good (> 95%) but failed to achieve viral suppression (n = 49). (DOCX 13 kb) [file 41182_2019_135_MOESM1_ESM.docx]

**Supplement table**

**Key characteristics of adolescents whose adherence level was good (>95%) but failed to achieve viral suppression (n=49)**

| **Characteristic** |  | **n** | **%** |
| --- | --- | --- | --- |
| Sex/Gender |  |  |  |
|  | Male | 31 | 63.3 |
|  | Female | 18 | 36.7 |
| Age |  |  |  |
|  | 10-14 years | 34 | 69.4 |
|  | 15-19 years | 15 | 30.6 |
| Years on ART |  |  |  |
|  | 0-2 years | 5 | 10.2 |
|  | 3-5 years | 16 | 32.7 |
|  | More than 5 years | 28 | 57.1 |
| Treatment interruption | | | |
|  | Yes | 7 | 14.3 |
|  | No | 42 | 85.7 |
| CD4 count at Initiation (cells/mL) | | | |
|  | More than 500 | 6 | 12.2 |
|  | 200-499 | 10 | 20.4 |
|  | less than 200 | 18 | 36.7 |
|  | missing | 15 | 30.6 |
| History of treatment Failure | | | |
|  | Yes | 14 | 28.6 |
|  | No | 35 | 71.4 |
| Regimen |  |  |  |
|  | AZT+3TC+NVP | 28 | 57.1 |
|  | AZT+3TC+EFV | 20 | 40.8 |
|  | ABC+3TC+EFV | 1 | 2.1 |
| Nutritional Status | |  |  |
|  | Normal | 45 | 91.8 |
|  | Severe/Moderately malnourished | 4 | 8.2 |
| Clinical Stage |  |  |  |
|  | I | 40 | 81.6 |
|  | II | 1 | 2.0 |
|  | III | 8 | 16.4 |
|  | IV | 0 | 0 |

Notes: Total sample is 49 adolescents. All other adolescents except 7 who experienced treatment interruption had an adherence of 100%.
